# Efficacy of Bimodal High-Voltage Monopulsed Current in the Treatment of Pressure Ulcer: A Systematic Review

**Published:** 2019-11

**Authors:** Zhiwei ZHANG, Bojun LI, Zhichao WANG, Lina WU, Lili SONG, Yexiang YAO

**Affiliations:** 1. Department of Nursing, The Second Affiliated Hospital of Qiqihar Medical University, Qiqihar, Heilongjiang Province, China; 2. The Second Clinical Medical College, Nanchang University, Nanchang, Jiangxi Province, China; 3. Department of Academic Theory Research, Qiqihar Medical University, Qiqihar, Heilongjiang Province, China; 4. School of Medical Technology, Qiqihar Medical University, Qiqihar, Heilongjiang Province, China; 5. Department of Anesthesiology, The Third Affiliated Hospital of Qiqihar Medical University, Qiqihar, Heilongjiang Province, China; 6. Department of Social Medicine and Health Management, School of Public Health, Qiqihar Medical University, Qiqihar, Heilongjiang Province, China

**Keywords:** High voltage pulse current, Pressure ulcer, Meta-analysis

## Abstract

**Background:**

We aimed to systematically evaluate the efficacy of high-voltage pulsed current (HVPC) in the treatment of pressure ulcer.

**Methods:**

We searched the databases of PubMed, Cochrane Library, Elsevier and EMBASE to identify randomized controlled studies on the application of HVPC in pressure ulcer treatment, up to January 2019. Two authors independently screened the literature according to the inclusion and exclusion criteria, extracted the data and evaluated the quality. RevMan 5.3 software was used for statistical analysis. Four randomized controlled trials involving a total of 176 patients were included in the study.

**Results:**

Meta-analysis showed that the percentage of wound area reduction in the HVPC treatment group was higher than that in the control group (95%CI 24.59, 47.76, *P*<0.001). Descriptive analysis showed that there was no significant difference in wound healing between the HVPC treatment group and the control group. One study reported that there was contact dermatitis, and the rest of the studies reported no adverse events.

**Conclusion:**

Compared with the conventional therapy, the combination with HVPC therapy can reduce the area of pressure ulcers more effectively. However, due to the small number of the studies included in this evaluation, the conclusions need to be verified by more high-quality studies.

## Introduction

Pressure ulceris defined as the damage caused by pressure or shear and/or friction on any type of skin. The main cause is the long-term application of external force at the bony ridge, leading to soft tissue ischemia and eventually causing necrosis (1). Although pressure ulcer can be prevented in most cases, it may affect rehabilitation, disable the patients to work or learn normally, disrupt the community reintegration, and ultimately influence the quality of life of the patients. When the condition is serious, it can lead to weakened mobility, loss of independence, surgical intervention if necessary, and the possibility of fatal infection (2).

The prevention and treatment of pressure ulcer is a major challenge in the rehabilitation and nursing care of long-term bedridden patients. Electrical stimulation technology has been used in the healing of chronic wounds for decades, and has made some progress (3,4). The current range for wound therapy studies includes low-intensity direct current (LIDC; <1.0 mA), microampere current (ultralow sub-sensory currents simulating natural current), low-voltage biphasic pulsed current (LVBPC), low-voltage monophase pulsed current (LVMPC) and high-voltage monophase pulsed current (HVMPC). Among them, the high-voltage pulsed current (HVPC) therapy is defined as the monophase bi-peaked current generated at a voltage of 75–200V and a frequency of 80–100 Hz, and the total current is generally 2.5 μA. Houghton (5) systematically evaluated 32 clinical studies of LIDC, microampere current, LVBPC, and LVPPC. Polak et al (6) also critically reviewed the role of HVMPC in chronic trauma. These authors believe that the induced wound healing depends on the electrical stimulation pattern and the particular method used, but the optimal parameters for stimulation and the electrical stimulation strategy for chronic wounds need to be further elaborated.

In order to study the safety and effectiveness of HVPC therapy in clinical practice, we evaluated the efficacy of HVPC for pressure ulcer by systematic evaluation and meta-analysis of the searched randomized controlled trials (RCTs).

## Methods

### Inclusion and exclusion criteria

Inclusion criteria were: 1) studies of electrical stimulation therapy used for treatment; 2) studies applied the electrical stimulation electrodes to the wound or around the surface of the wound; and 3) RCTs.

The exclusion criteria were: 1) electromagnetic field study; 2) studies used wound internal electrodes; 3) studies with more than 50% of data about ulcers of other causes (such as diabetic ulcers, and deep vein thrombosis); 4) the outcome indicators reported were ambiguous, which was ineligible for the data merger; 5) studies in which the intervention methods were unclearly described ; 6) studies with obvious data error and mistakes in the use of statistical methods; 7) repetitive studies; and 8) animal experiments.

### Subjects

All the pressure ulcer of any cause and severity, patients aged over 18 years, not limited in race, age, gender and duration of disease.

### Interventional methods

HVPC combined with standard therapy was used in the experimental group; standard therapy, including debridement, drug dressing, nutritional support, physical and occupational therapy, was used in the control group, with or without pseudo-current therapy.

### Outcome indicators

The main outcome indicators is the percentage of wound area reduction. For initial wound area calculation, transparent sulfuric acid paper was applied to the skin surface, and the range of pressure ulcer was drawn on the paper according to the ulcer boundary. The picture was processed in image processing software (Photoshop) to calculate the area (cm^2^). The percentage of wound area reduction = (initially treated wound area - end-treatment wound area) / initially treated wound area × 100%. The secondary outcome indicators included: condition of wound healings (ulcer healing and crust falling off); and presence of adverse reactions.

### Search strategy

The PubMed, Cochrane Library, Elsevier, EM-BASE and other databases were searched by two researchers using the terms [(pressure ulcer OR pressure sore) AND (high-voltage pulsed current OR high-voltage pulsed stimulation OR twin peak monophasic)] to identify the RCTs on the application of HVPC therapy in pressure ulcer, published up to January 2019. After the search was completed, cross-checking was performed. Any disagreement was resolved via discussion and, if necessary, a third researcher joined in the discussion for a final consent.

### Literaturequality evaluation

The quality evaluation of the searched literature was performed using the risk of bias assessment form the Cochrane Collaboration, and a table of the results was generated. The evaluation of “low risk”, “unknown risk” and “high risk” was determined according to the checklists in the table. Seven items were used in the evaluation: the random sequence generation method; the allocation concealed scheme; the blind method of the subject and the tester; the blind method of the test results; evaluation of incomplete data, selective reporting; and other biases. Any disagreement in the process of the evaluation was resolved according to the scoring table, or a third researcher was invited to join in the discussion.

### Statistical analysis

Systematic evaluation and meta-analysis were performed using RevMan 5.3 software. The odds ratio (OR) was used as the quantitative analysis index of the count data, while the measurement data was evaluated by the mean difference (MD), and 95%CI was also included in the evaluation index. Included studies were tested by Chi-square test to verify its heterogeneity. When *I*^2^>50% or *P*<0.1, the heterogeneity of the included studies was significant, and descriptive analysis was then performed to find the causes of heterogeneity using the random effect model. When *I*^2^≤ 50% or *P*≥0.1, the heterogeneity was acceptable, and the fixed effect model was used.

## Results

### Included articles

**Fig. 1:**
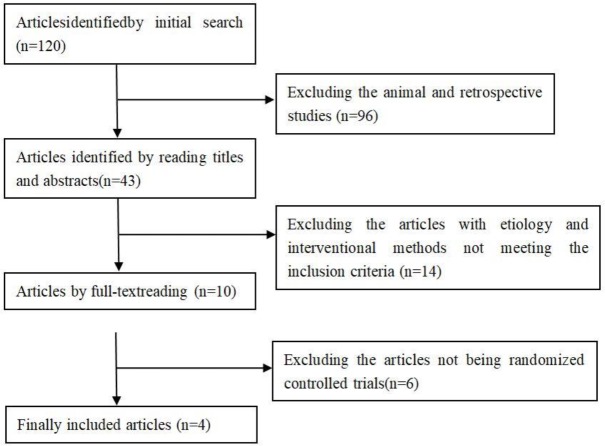
The literature screening process

A total of 120 articles were identified after the literature searching. Based on preliminary judgment, 96 articles of animal and retrospective studies did not meet the inclusion criteria. By reading the titles and abstracts, 14 articles did not meet the inclusion criteria in term of etiology and interventional methods (7–20), and the full texts of the remaining 10 articles (21–30)were read, and finally four RCTs (21–24) were included in the analysis. The entire literature searching process is shown in Fig. 1.

### Characteristics of the included studies

The included four RCTs were published between 2010 and 2017, with a total of 176 patients (92 in the HFPU therapy group and 84 in the control group). The baseline levels were consistent between the RCTs (including the age of the patients, gender, disease type, and severity of pressure ulcer) (all *P*>0.05). However, the case numbers of the four RCTs included were small and all were small sample studies. Other basic characteristics of the included studies are shown in Table 1.

**Table 1:** Basic characteristics of the included studies

***Reference***	Age (yr)^*^	***Gender (M/F)***	***Pressure ulcer stage▲***	***Cases/affected areas***		***Interventions***		***Treatment methods***		***Outcome indicators***

				***T***	***C***	***T***	***C***		***Treatment days***	
(21)	T: 50.3±17.3 (23–74) C: 50.8±11.6 (32–79)	20/14	II, III, IV, X	16	18	Electrical stimulation therapy + standard therapy: bimodal monophase pulse current, stimulation frequency 100Hz /10 Hz/0Hz, 20 min for each cycle, voltage 50–150V	Standard therapy ※	1 time a day, 8 hours each time	90	①②
(16)	T: 79.92±8.50/60–92/80 C: 76.33±12.74/60–95/81	37/10	II, III	25	24	Electrical stimulation + standard therapy: bimodal single-phase pulse current, stimulation frequency 100 Hz, voltage 100 V	Standard therapy + no current therapy	5 times a week, 50 mineach time	42	①②
(17)	T: 79.35±8.48 C: 77.55±12.24	52/11	II, III, IV	23	20	Electrical stimulation therapy + standard therapy: bimodal single-phase pulse current, stimulation frequency 100Hz, voltage 100V	Standard therapy + no current therapy	5 times a week, 50 min each time	38	①②
(24)	T: 59.0±18.16 C: 56.2±19.70	22/28	II, III	26	24	Electrical stimulation therapy + standard therapy: bimodal single-phase pulse current, stimulation frequency 100Hz, voltage 100	Standard therapy	1 time a day, 50 min each time	42	①

Note: T, observation group; C, control group;

* mean±SD / age range / average age;

▲ evaluation criteriafor pressure ulcer stage;

※ standard therapy, including debridement, drug dressing, nutritional support, physical and occupational therapy

### Evaluation of methodological quality of the included studies

**Fig. 2:**
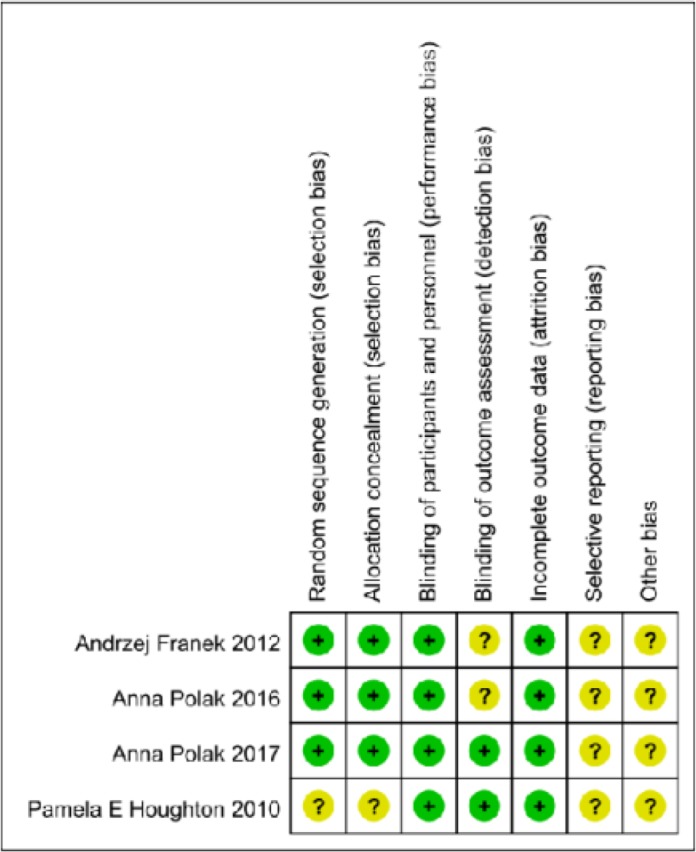
Quality evaluation of the included studies

The evaluation was conducted using the risk of bias analysis form the Revman 5.3 software on the Cochrane website and according to its description of the requirements (Fig. 2). There were three high-quality studies (22–24), which described in detail the randomization scheme, the allocation concealment scheme and the blinding method, and in these studies, the wound surface was all covered with gauze to prevent the pseudo-current of the electrode. One article was of lower quality (21), in which the randomized protocol was not described in detail and no placebo therapy was used. All studies described the status of loss to follow-up or patients’ withdrawal from the studies.

### Evaluation of treatment outcomes Percentage of wound area reduction

**Fig. 3:**
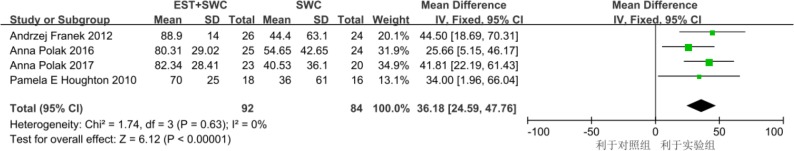
Percentage of wound area reduction

The percentage of wound area reduction was described in all the four RCTs, and their heterogeneity was small (*P*=0.63, *I*^2^=0%). The results showed that electrical stimulation therapy plus standard therapy was more effective in treating pressure ulcers than the therapy used in the control group (95%CI 24.59, 47.76, *P*<0.001)(Fig. 3).

### Wound healing

Three RCTs (21–23) reported complete healing of the wound at the end of treatment. Due to the small number of samples in the study and the large difference in outcome between the groups, and the significant clinical heterogeneity, these 3 RCTs underwent descriptive analysis (Table 2). Two studies (21,22)showed that the difference was not significant in comparison of the effect of the HVPC therapy in promoting complete wound healing, thus a subgroup analysis was performed. In a study (21), there were 4 cases and 1 case of grade II pressure ulcer in the control group and the experimental group, respectively. As grade II pressure ulcer can achieve a 100% complete healing, the outcome indicators were influenced.

**Table 2:** Descriptive analysis of the three RCTs

***Reference***	***Clinical trial stage***	***Complete wound healing***		**P *value***
		***HVMP***	***Control group***	
(22)	II	9/11	6/11	0.74
	III	3/14	1/13	0.6
(23)	II, III, IV	11/23	0/20	0.013
(21)	II	1/1	4/4	0.62
	III, IV, X	5/15	1/14	0.55

### Adverse events

One study (21) reported occurrence of contact dermatitis, and the rest of the studies reported no adverse events.

## Discussion

Pressure ulcer is the most common complication in long-term bedridden patients, and its prevention and treatment have been studied for a long time. Electrical stimulation technology is currently considered an effective adjuvant to standard therapy for pressure ulcer, which can accelerate the healing of chronic wounds (31). The pulse current for clinical treatment can be divided into low-voltage and high-voltage pulsed currents depending on the voltage at which the pulse current is generated. Previous studies have systematically evaluated and meta-analyzed the therapeutic effects of electrical stimulation therapy in patients with spinal cord injury and pressure ulcer(32), compared the effects of the electrical stimulation therapy with the standard therapy, and found that the area of pressure ulcer was reduced by 1.32% each day (95%CI: 0.58–2.05, *P*<0.001). However, this study did not classify different types of electrical stimulation therapy, and did not provide more adequate evidence-based medical data for the efficacy of HVPC therapy. The present study used systematic evaluation and meta-analysis to evaluate the RCT using HVPC to treat pressure ulcer.

This systematic analysis included four RCTs to evaluate the efficacy of HVPC therapy in the treatment of pressure ulcer. Houghton et al (21)found that the reduction rate (70±25%) of the pressure ulcer wound surface area (WSA) in the combined treatment group was significantly higher than that in the SWC group (36±61%; *P*=0.048). For moderate to severe pressure ulcers, such as stages III, IV or X, the ratio of greater than 50% in the reduction of pressure ulcer WSA in HFPC therapy group was significantly higher than that in the SWC group (*P* = 0.02).

In addition, WSA was significantly decreased after 1 week of intervention in the HVPC therapy group (*P*=0.032) (22). At the 6th week after treatment, the percentage of WSA reduction in the treatment group was 80.31±29.02%, and that in the control group was 54.65%±42.65% (*P*=0.046). Polak et al (23) again compared the use of anode and cathode HVPC therapy. In their study, the results of anode use were extracted and analyzed. Franek et al (24) also demonstrated a significant decrease in wound area and linear measurements (*P*<0.05) and an increase in granulation tissue (*P*=0.006) in the HVPC therapy group. From the second week of treatment, the changes of wound surface area, linear measurement, wound volume and granulation tissue in the treatment group were significantly greater than those in the control group. The change of surface area at the sixth week was 88.9%±14%, and that of the control group was 44.4% ± 63.1% (*P*=0.00003). Combining the reduction percentage in pressure ulcer area in the included studies, the meta-analysis showed that HVPC therapy combined with standard therapy was more effective in treating pressure ulcer than standard therapy alone. Except for the contact dermatitis reported in the included literature, no other serious adverse events were reported.

There were some limitations in this study. 1) Some unpublished “grey” literature might be missed during the inclusion process, resulting in insufficient data inclusion. Two of the included articles were by a same author working with different centers (22,23), so there might be biases of data in multiple publication.

2)The small number of included studies may lead to selective bias and factual bias.

3) In the included literature, the pressure ulcer classification focused onstage II–IV pressure ulcers, yet in some studies, the outcome indicators were not described for different stages, which made subgroup analysis impossible tojudge whether HVPC therapy has a significant impact on different stages of pressure ulcers.

## Conclusion

The combination of HVPC therapy with standard therapy can significantly reduce thepressure ulcer wound area, and the efficacy of the HVPC therapy combined with standard therapy for pressure ulcer was better than that of the standard therapy alone. However, due to the small number of high-quality studies, the conclusions need to be verified by more high-quality RCTs, thus providing more evidence for clinicians to select the optimal treatment for this condition.

## Ethical considerations

Ethical issues (Including plagiarism, informed consent, misconduct, data fabrication and/or falsification, double publication and/or submission, redundancy, etc.) have been completely observed by the authors.
